# Development and Effectiveness of a Community Capacity Building Program for the Wellness of Traditional Marketplace Merchants: A Pilot Study

**DOI:** 10.3390/ijerph182212238

**Published:** 2021-11-22

**Authors:** Yeojoo Chae, Yeongmi Ha

**Affiliations:** 1 Department of Nursing, Changwon Moonsung University, Changwon 51410, Gyeongsangnam-do, Korea; yjchae413@hanmail.net; 2 Institute of Health Sciences, School of Nursing, Gyeongsang National University, Jinju 52727, Gyeongsangnam-do, Korea

**Keywords:** traditional marketplace, leadership, self-efficacy, wellness, community capacity

## Abstract

Merchants in the traditional marketplace are exposed to poor working conditions, such as long working hours, musculoskeletal stress, lack of physical activity, irregular meal times, and nutritional imbalance. This study aimed to develop a community capacity-building program for the wellness of traditional marketplace merchants and examine its effectiveness. A total of 60 merchants from two traditional markets were allocated to an experimental group and control group. The program consisted of four sections: Training wellness leaders in the traditional marketplace, wellness, organizing wellness committee, and promoting wellness partnerships. The program was conducted over 12 weeks. Significant differences were observed in week 12 between the experimental and control groups in leadership (*p* = 0.010), health knowledge (*p* < 0.001), health self-efficacy (*p* = 0.016), wellness (*p* = 0.001), and community capacity (*p* = 0.038). The community capacity-building program for the wellness of traditional marketplace merchants was effective in improving marketplace merchants’ leadership, health knowledge, health self-efficacy, wellness, and community capacity. Therefore, it is recommended to actively utilize this community capacity program for market merchants.

## 1. Introduction

It is well known that merchants in the traditional market are exposed to poor working conditions, such as long working hours, musculoskeletal stress, lack of physical activity, irregular meal times, and nutritional imbalances [1]. The average age of Korean merchants in the traditional market as of 2018 was 56.8 years and the average daily working time was 10.4 h, indicating that they spend most of the day in their stores and markets [2]. Hypertension and diabetes were higher among merchants in the traditional market than a national representative sample of Koreans [3]. Merchants in the traditional market in Korea tend to be more affected by chronic diseases and are likely to be vulnerable due to poor working environments [3]. As merchants spend many hours of the day at a workplace, an effective wellness program should be established in the traditional market.

The World Health Organization (WHO) encourages setting-based approaches, such as schools, workplaces, marketplaces, towns, and cities where people live to promote health over personal approaches [4]. Healthy settings for wellness involve multidisciplinary approaches that integrate action across risk factors. The key components of a setting-based approach to health promotion are community building and participation, sustainable community resources, and education on health issues. Korea joined the Federation of Healthy Cities in 2004 and established various setting-based health programs, such as healthy cities, healthy rural villages, and health-promoting hospitals [5]. Despite increased attention, a healthy marketplace program to pursue the sustainable wellness of merchants in the traditional market has not yet been initiated in Korea.

When the community is treated as a healthy setting, communities can offer a resource for changing individual health behaviors [6]. Community-based interventions, such as community capacity building, have been developed for health promotion building within the community as a healthy setting [6]. Although the concept of community capacity-building may differ among scholars, community capacity is defined as the interaction of human capital, organizational resources, and social capital within a community, which can be used to solve the population’s problems and improve the community’s wellbeing [6]. Previous community capacity-building programs included combining leadership training and organizational development to promote the wellness of black people [7], community leadership training in a healthy city [8], obesity prevention and environmental changes among low-income people [9], and health promotion projects in rural areas [10]. However, community capacity-building programs to create a healthy market for merchants are lacking. Establishing a healthy traditional marketplace requires collective effort to promote health among individuals and organizations, including individual capacity-building and efforts to reinforce a traditional healthy marketplace. Therefore, this study aimed to develop a community capacity-building program for the wellness of merchants in a traditional marketplace and examine its effectiveness.

## 2. Materials and Methods

### 2.1. Study Design

This was a quasi-experimental study using a pre- and post-design with a non-equivalent control group to verify the effectiveness of a community capacity-building program for the wellness of merchants in a traditional marketplace. One non-randomized group received the intervention for 12 weeks, and the control group received the usual health promotion program. The variables of leadership, health knowledge, health self-efficacy, wellness, and community capacity were measured at baseline and after 12 weeks. 

### 2.2. Participants

The participants were merchants in two traditional markets, the J and G markets, recruited using convenient sampling. The inclusion criteria were being a store merchant and being able to read and understand the contents of the workbook provided by the program. The exclusion criteria were being a street merchant and suffering from cardiovascular or musculoskeletal diseases that prevented participation in the wellness program. 

The participants were divided into two groups, with the J market being an experimental group and the G market a control group. J and G were similarly-sized traditional markets situated in J city. The two markets mainly engaged the wholesale and retail of similar goods, and the merchants in the two markets had similar socioeconomic statuses and working conditions. A recruitment notice regarding participating in a community capacity program was placed on the market bulletin board two weeks before the start of the program. Those who wished to participate in the study were included on a first-come, first-served basis if they met the inclusion criteria. For the convenience of the researcher, participants in the J market were designated as the experimental group, and a control group was recruited from the G market using a matching technique that considered the key characteristics of the experimental group. The experimental group participated in a community capacity-building program for healthy marketplaces, and the control group participated in a traditional health promotion program.

The sample size was estimated using the G*power 3.1.9 program [11]. The *t*-test demonstrated a power (1 − β) of 0.80, significance level (ɑ) of 0.05, and effect size of the independent *t*-test (d) of 0.50. The minimum number of samples for each group was determined to be 27 people. Considering the 10% dropout rate during the intervention, 60 people were selected as 30 experimental groups and 30 controls. 

### 2.3. Measurements

#### 2.3.1. Leadership

Leadership was assessed using a servant leadership measure [12] after translation into Korean. This instrument was composed of 28 items rated on a five-point Likert scale (1 = completely disagree, 5 = completely agree), where higher scores indicate higher servant leadership. Cronbach’s alpha for this study was 0.92. 

#### 2.3.2. Health Knowledge 

The research team developed an instrument for promoting health knowledge. A preliminary instrument was developed based on the current scientific literature on hypertension [13], diabetes [14], and osteoporosis [15], which are prevalent among Korean marketplace merchants. The final items were selected through the verification of content validity by 3 experts, 2 nursing professors, and a head nurse with more than 10 years of clinical practice experience. The content validity index (CVI) was calculated by dividing the sum of the scores of 3 experts for each item by the total score when each expert gave the highest score of 4 points. An item was determined to have content validity if the CVI was 0.8 or higher [16]. Only items with a CVI of 0.8 or higher were selected through two revisions after expert review. A total of 24 items were selected.

The instrument consisted of three parts: Hypertension, diabetes, and osteoporosis. Each part contained 8 items, with a total of 24 items. The contents consisted of definition, symptoms, exacerbation factors, treatment, and complications, which were scored by assigning one point for each correct answer. The total score ranged from 0 to 24, with higher scores indicating higher health knowledge. The Kuder and Richardson Formula 20 (KR20) in this study was 0.80.

#### 2.3.3. Health Self-Efficacy

Health self-efficacy [17] was used to measure health practice abilities after translation into Korean. This instrument consisted of 28 items rated on a four-point Likert scale (1 = completely disagree, 4 = completely agree). The total score ranged from 28 to 112, with higher measurement scores indicating higher health self-efficacy. Cronbach’s alpha for this study was 0.91.

#### 2.3.4. Wellness

Wellness was measured using the wellness index for workers [18]. This instrument consisted of 18 items rated on a five-point Likert scale (1 = completely disagree, 5 = completely agree), with higher scores indicating higher wellness. Cronbach’s alpha for this study was 0.89.

#### 2.3.5. Community Capacity

The Korean version of the community capacity-building tool (CCBT) [19] was used in this study. This instrument was composed of 28 items rated on a four-point Likert scale (1 = just starting, 2 = progressing, 3 = doing alright, 4 = doing well). Higher scores indicated higher community capacity. Cronbach’s alpha for this study was 0.93.

### 2.4. Community Capacity-Building Program

#### 2.4.1. Development of Community Capacity-Building Programs for Traditional Market Merchants

The community capacity-building program for traditional market merchants was based on four strategies for strengthening community capacity, as suggested by Chaskin et al. [6]: Leadership development, organizational development, community organizing, and fostering collaborative relations among organizations. The community capacity-building program for traditional market merchants aimed to cultivate wellness leaders in traditional marketplaces to improve leadership and wellness programs to improve health problem-solving abilities, develop a wellness committee to increase community awareness, and promote wellness partnerships to increase access to resources.

1. Training wellness leaders in traditional marketplaces

The purpose of training wellness leaders in the traditional marketplace was to promote leadership. This aimed to engage the participation and commitment of current and potential leaders, provide them with opportunities to enhance leadership skills, connect them to new information and resources, broaden their perspectives on their community and its development, and help them build relationships [6]. The training contents consisted of educational information and skills related to hypertension, diabetes, and osteoporosis. The program included one orientation session, three physical activity sessions, two hypertension management sessions, two diabetes management sessions, two osteoporosis management sessions, one nutrition management session, and one SWOT analysis session to create healthy and happy traditional marketplaces. To fully understand the educational content of each session, lectures and practice were organized in parallel. Before classes, the researcher and participants set long- and short-term goals together. The practice classes (2nd, 3rd, 5th, 7th, and 9th session) were first performed by the researcher, followed by the participants directly after observation, providing a fulfilling experience. After class, assignments were distributed to merchants, and feedback on behaviors and feelings when performing the assignments was collected in the following class to further strengthen training. A workbook on effective self-care of health-promoting behaviors was distributed. The contents were organized in an easy-to-follow way with pictures for easy use in the workplace. In addition, the concept, goal setting, and assignments were directly written to allow the participants to familiarize themselves with the contents at any time. The workbook contents were divided into conceptual and practice sections, and the subjects consisted of hypertension, diabetes, and osteoporosis. Conceptual contents of the treatment and prevention of hypertension, diabetes, and osteoporosis were presented first. Practice, such as blood pressure and blood glucose measurement methods, were explained following this. Next, nutrition management and exercise methods for prevention were presented. In addition, a SWOT analysis was conducted on the market.

2. Wellness program

The purpose of wellness programs was to develop health problem-solving skills related to community capacity strategies. The wellness program aimed to develop community organizing, broadly bringing people together to solve community problems and address collective goals [6]. The program was based on Hettler’s [20] six wellness concepts and structured around topics that merchants could participate in and wanted based on preliminary research. It consisted of aerobic exercise to promote physical wellness, recreational and healing meditation to promote emotional, spiritual, and occupational wellness, and club activities to promote cognitive and social wellness.

Aerobic exercise, such as line dance (60 min per session, 12 times), recreation, such as laughter therapy (60 min, one time), club activities, such as learning to wrap a gift and write calligraphy (60 min per session, seven times), and healing meditations (10 min, 12 times) were used to help manage stress efficiently.

3. Organizing a wellness committee

Organizing a wellness committee aimed to help marketplace merchants be aware of a sense of community and enhance community competencies. Organizational development creates a new organization in the community or reinforces existing organizations to perform a new function or role to have an integrated connection [6]. The wellness committee included six people. The wellness committee was created to promote the health of marketplace merchants and agreed to conduct various programs, such as training for wellness leaders and wellness programs. The wellness committee held three meetings on the wellness of traditional marketplace merchants.

4. Promoting wellness partnerships

Wellness partnerships aimed to promote collaborations, partnerships, and organizational networks among community competencies. Fostering collaborative relationships among organizations often focuses on the organizational infrastructure, seeking to change the ways individual organizations relate to one another and to organizations and actors beyond the neighborhood [6]. To promote wellness partnerships, three health booths were installed with personnel from the health promotion team at a public health center, with contents on smoking cessation (first round), low alcohol consumption (second round), and infectious disease prevention and dental health (third round). In addition, blood pressure, blood sugar, BMI measurements, and customized health counseling were provided.

#### 2.4.2. Implementation of Community Capacity-Building Programs

This study was approved by the Institutional Review Board of Gyeongsang National University in South Korea (GIRB-A18-Y-0053). Participants were given information on the study purpose and methods and were informed that participation was voluntary. Confidentiality of personal information was assured and participants were informed that they could stop it at any time if they wished during the program. Informed written consent was obtained from all participants prior to participation.

Interventions conducted in the experimental group consisted of four sections: Training wellness leaders in the traditional marketplace, conducting wellness programs, organizing wellness committees, and promoting wellness partnerships. The training of wellness leaders in traditional markets consisted of 12 sessions over 12 weeks. The wellness promotion program consisted of programs to enhance its components and was conducted in 31 sessions over 12 weeks. The time for each session was different. The organizational development of the wellness committee consisted of two wellness committee meetings. Promotion of the wellness partnership was conducted three times over 12 weeks. The health booth and health consultation were conducted three times.

### 2.5. Statistical Analyses

The collected data were analyzed using SPSS WIN 24.0. First, the participants’ general characteristics were analyzed using the average standard deviation of the frequency percentage. Second, the Shapiro–Wilk test was used to verify the normality of the study variables in the experimental and control groups. Third, a homogeneity test was conducted using a chi-square test with Fisher’s exact test and an independent *t*-test. Fourth, the effectiveness of the intervention on leadership, health knowledge, health self-efficacy, wellness, and community capacity was examined using the paired *t*-test, independent *t*-test, and Mann–Whitney U test.

## 3. Results

### 3.1. Homogeneity of General Characteristics in Participants

There were no significant differences in the general characteristics between the two groups (Table 1). The average age was 63.10, with the largest group aged over 65 years (48.3%), followed by 55 to 65 years (40.0%). Most participants were married (63.3%). Most participants were high school graduates (41.7%). Wholesale and retail businesses accounted for the majority (60.0%). The average daily working time was 10.37 h. Subjective health status was moderate (55.0%).

### 3.2. Homogeneity of Study Variables between Two Groups

There were no significant differences in the dependent variables of leadership, health knowledge, health self-efficacy, wellness, and community capacity (Table 2).

### 3.3. Effectiveness of Intervention

There were statistically significant differences between the two groups in leadership (z = −2.59, *p* = 0.010), health knowledge (*t* = 4.92, *p* < 0.001), health self-efficacy (*t* = 2.47, *p* = 0.016), wellness (*t* = 3.68, *p* = 0.001), and community capacity (*t* = 2.12, *p* = 0.038; Table 3).

## 4. Discussion

As traditional marketplace merchants face difficulty in engaging in health promotion activities due to poor working conditions, wellness programs based on healthy setting approaches are crucial. The average age of the participants in this study was 63.10 years old, accounting for 88.3% of the total. Over 60% of the participants were engaged in wholesale and retail business, and the average daily working time was 10.37 h, indicating that they spent most of the day in marketplaces. In addition, poor working conditions, such as long working hours and characteristics of a one-person business, prevented market merchants from participating in wellness programs provided by public health centers far from traditional marketplaces. As many participants were middle-aged or old, healthcare is important. This indicates the need for a customized program using healthy setting approaches targeting merchants in traditional marketplaces.

This study revealed significant differences in leadership scores between the experimental and control groups after participation in the community capacity-building program for traditional market merchants. Goodman et al. [21] emphasized that participation and leadership are closely related. To maximize participation and leadership, it is necessary to engage people’s abilities and provide opportunities for collective leadership [21]. This study encouraged supporting the growth and success of community members by sharing the roles of each individual within the small group. After consulting with the team members, wellness leaders for hypertension, diabetes, and osteoporosis were selected and delegated authority. During the practice class, each leader was facilitated during practice. Among the programs that the subjects of this study participated in, leadership lectures for leaders and SWOT analysis of traditional markets were considered to have helped cultivate a sense of community and increase leadership. In addition, they can strengthen members’ commitment to the constituent concepts of community capacity by increasing leadership.

Health knowledge scores in the experimental group significantly increased after the program, while there were no significant differences in the control group. This is in line with previous studies that indicated that higher knowledge regarding diseases or symptoms resulted in higher transition to preventive or health promotion actions [22,23]. In small groups, education methods considered the subjects’ age and characteristics, and operation methods, such as sphygmomanometers and blood glucose meters, were demonstrated and practiced. The effect could be increased by working in groups of two and freely expressing opinions. During the program, the participants used the workbook, and organized the contents to participate and write a theoretical explanation, including pictures of diseases by subject, checklists by behavior during practice, contents about nutrition, and organization at the end of the unit. In addition, a quiz was provided at the end of each theory class, allowing the participants to review and increase their health knowledge. Therefore, this program provided knowledge for health and wellness leaders. It is hoped that this program will become a foundation for the healthy lives of traditional market merchants by spreading knowledge to nearby merchants in the market. In addition, increasing the health knowledge of traders in traditional markets should improve the ability to solve health problems, which is a constituent concept of community capacity.

Health self-efficacy scores of the experimental group significantly increased after the programs, while there were no significant differences in the control group. This is in line with Foster et al. [24], who used an eight-week health program targeting community residents and found that health self-efficacy increased after intervention. Health self-efficacy refers to the level of self-confidence perceived as performing general health-promoting behaviors and consists of exercise efficacy, psychological efficacy, nutritional efficacy, and health management efficacy [17]. This study included the theoretical content on hypertension to enable efficient health management, and exercise efficacy was improved through stretching to prevent hypertension, followed by a diet suitable for hypertension. Nutritional and psychological efficacy improved through healing meditation. Including theoretical content on each topic, exercises, and psychological and nutritional content helped improve health self-efficacy. In addition, practice provided the experience of achievement through support among colleagues, praise for performing correct movements, and encouragement through verbal persuasion. Improving self-efficacy by allowing members to maintain a relaxed state when practicing as a group improved the health self-efficacy of traditional market merchants, thereby enhancing their ability to solve health problems.

The wellness scores of the experimental group significantly increased after the program, while there were no significant differences in the control group. Previous studies using the concept of community capacity building [19,25] demonstrated that quality of life increased significantly after the health promotion program. In this study, to promote the wellness of the traditional market, a program was provided based on the sub-concept of wellness. Line dancing promoted physical wellness, and club activities promoted cognitive wellness and social wellness. In addition, laughter therapy and healing meditation enabled traditional market merchants’ emotional and spiritual wellness.

Community competency scores of the experimental group significantly increased after the program, while there were no significant differences in the control group. Although previous studies using community competency as a measurement variable were scarce, Fletcher et al. [26] demonstrated the effect by applying a drug and alcohol prevention program for indigenous peoples in Canada. Unlike previous studies that used only a part of strategies or constituent concepts, this study used all four strategies to reinforce the four constituent concepts of community capacity proposed by Chaskin [27]. To strengthen community capacity, it is important to increase the capacity of members in the community, the degree of convergence between the organization and individuals, and the skills to properly utilize resources inside and outside the organization. It is crucial to use strategies that strengthen the entire concept of community capacity.

The meaningful finding of this study is that effect was extended from the individual residents of the community to the entire community. After the program was terminated, merchants in the experimental market opened line dance classes and were planning to start other programs to promote the health and happiness of traditional market merchants. Community capacity is the basis for assessing local residents’ competency levels and the strength between community organizations [28]. As the leaders of the community commit themselves to the community and are organized and expanded, the environment of the community will gradually change, allowing true ‘healthy settings’ to be realized. Therefore, it is important to note that the participants’ wellness improved through a wellness program that can be used in business sites, despite the poor working environment and difficulty in conducting activities outside of working hours. Therefore, rather than providing a fragmentary health promotion program for groups that have difficulty receiving institutional benefits, local community capacity was reinforced, allowing for autonomous health promotion. In addition, there is a need to improve policies and systems for this.

This study had some limitations. First, as this study was conducted with a traditional market located in J city, a small-sized city, the results cannot be generalized to the entire traditional market of Korea. Second, owing to the small proportion of a relatively homogenous group of merchants from one traditional marketplace, findings of main outcomes may not be generalizable to all types of traditional marketplace merchants. Future studies should examine community capacity-building programs considering various characteristics of traditional markets and targeting large cities and other small- and medium-sized cities. Third, future studies should measure the persistence of effects through follow-up surveys 6 and 12 months after program implementation to determine the continuous effectiveness of the local community capacity-building program for the wellness of traditional market merchants.

## 5. Conclusions

The community capacity-building program for traditional market merchants was effective in promoting leadership, developing health problem-solving skills, organizing wellness committees, and promoting wellness partnerships. The program improved the leadership, health knowledge, health self-efficacy, wellness, and community capacity of traditional marketplace merchants. Therefore, future research should focus on traditional market marketplace merchants and groups that cannot benefit from health promotion at an institutional and organizational level. In addition, public health workers should focus on strengthening their community capacity by providing community capacity building programs.

## Figures and Tables

**Table 1:** Homogeneity test of general characteristics.

Characteristics	Exp. (*n* = 30)	Cont. (*n* = 30)	*t*/(*p*)
	*n* (%)	*n* (%)	
Mean age (year) ± SD	62.50 ± 5.68	63.70 ± 7.38	−0.71 (0.483)
≤54	4 (13.3)	3 (10.0)	1.67 (0.434)
55–64	14 (46.7)	10 (33.3)	
≥65	12 (40.0)	17 (56.7)	
Marital status			
Single	1 (3.3)	1 (3.3)	0.39 (1.000) ^†^
Married	19 (63.3)	19 (63.3)	
Bereavement	8 (26.7)	9 (30.0)	
Divorce/separation	2 (6.7)	1 (3.3)	
Education level			
≤Elementary	3 (10.0)	9 (30.0)	4.04 (0.132)
Middle	12 (40.0)	11 (36.7)	
≥High	15 (50.0)	10 (33.3)	
Living conditions			
Alone	10 (33.3)	6 (20.0)	7.50 (0.058)
Couple	5 (16.7)	15 (50.0)	
Couple & Children	10 (33.3)	6 (20.0)	
Extended family	5 (16.7)	3 (10.0)	
Occupation			
Wholesale and retail	17 (56.7)	19 (63.3)	−0.32 (0.752)
Sewing	9 (30.0)	3 (10.0)	
Food service business	4 (13.3)	8 (26.7)	
Working hours (hour/day)	10.19 ± 1.51	10.57 ± 1.74	−0.89 (0.377)
Subjective health status	2.93 ± 0.74	3.03 ± 0.77	−0.52 (0.609)
Unhealthy	6 (20.0)	7 (23.3)	
Moderate	17 (56.7)	16 (53.3)	
Healthy	7 (23.3)	7 (23.3)	

Abbreviations: SD, standard deviation; Exp., Experimental group; Cont., Control group. ^†^ Fisher’s exact test.

**Table 2:** Comparison of study variables between groups at baseline.

Characteristics	Exp. (*n* = 30)	Cont. (*n* = 30)	*t*/(*p*)
	*n* (%)	*n* (%)	
Leadership	3.22 ± 0.51	3.27 ± 0.55	−0.38 (0.176)
Health Knowledge	13.73 ± 3.04	12.80 ± 4.29	0.97 (0.335)
Health Self-efficacy	80.23 ± 10.08	81.00 ± 12.83	−0.26 (0.798)
Wellness	3.36 ± 0.47	3.45 ± 0.67	−0.62 (0.538)
Community Capacity	2.72 ± 0.54	2.42 ± 0.66	1.94 (0.057)

Abbreviations: Exp., Experimental group; Cont., Control group.

**Table 3:** Effect of the community capacity building program.

	Group	Pre-Test	Post Test	Effect by Point	Intergroup Effect
		M ± SD	M ± SD	*t* (*p*) or z (*p*)	*t* (*p*) or z (*p*)
Leadership ^†^	Exp. (*n* = 30)	3.22 ± 0.51	3.63 ± 0.26	−3.57 (<0.001)	−2.59 (0.010)
	Cont. (*n* = 30)	3.27 ± 0.55	3.29 ± 0.55	−1.67 (0.095)	
Health knowledge	Exp. (*n* = 30)	13.73 ± 3.04	16.83 ± 3.05	6.77 (<0.001)	4.92 (<0.001)
	Cont. (*n* = 30)	12.80 ± 4.29	11.53 ± 3.99	−1.67 (0.106)	
Health self-efficacy	Exp. (*n* = 30)	80.23 ± 10.08	87.10 ± 8.09	3.15 (0.004)	2.47 (0.016)
	Cont. (*n* = 30)	81.00 ± 12.83	79.00 ± 15.91	−0.70 (0.488)	
Wellness	Exp. (*n* = 30)	3.36 ± 0.47	3.86 ± 0.35	4.81(<0.001)	3.68 (0.001)
	Cont. (*n* = 30)	3.45 ± 0.67	3.29 ± 0.70	−1.11 (0.278)	
Community capacity	Exp. (*n* = 30)	2.72 ± 0.54	3.12 ± 0.51	4.0 (<0.001)	2.12 (0.038)
	Cont. (*n* = 30	2.42 ± 0.66	2.49 ± 0.57	0.57 (0.572)	

Abbreviations: SD, standard deviation; Exp., Experimental group; Cont., Control group. ^†^ = Mann-Whitney U test

## Data Availability

The datasets generated and/or analyzed during the current study are not publicly available due to concerns regarding privacy, but select data are available from the corresponding author upon reasonable request.
